# The application of BH3 mimetics in myeloid leukemias

**DOI:** 10.1038/s41419-021-03500-6

**Published:** 2021-02-26

**Authors:** Narissa Parry, Helen Wheadon, Mhairi Copland

**Affiliations:** Paul O’Gorman Leukaemia Research Centre, University of Glasgow, Glasgow, UK

**Keywords:** Targeted therapies, Acute myeloid leukaemia, Chronic myeloid leukaemia

## Abstract

Execution of the intrinsic apoptotic pathway is controlled by the BCL-2 proteins at the level of the mitochondrial outer membrane (MOM). This family of proteins consists of prosurvival (e.g., BCL-2, MCL-1) and proapoptotic (e.g., BIM, BAD, HRK) members, the functional balance of which dictates the activation of BAX and BAK. Once activated, BAX/BAK form pores in the MOM, resulting in cytochrome c release from the mitochondrial intermembrane space, leading to apoptosome formation, caspase activation, and cleavage of intracellular targets. This pathway is induced by cellular stress including DNA damage, cytokine and growth factor withdrawal, and chemotherapy/drug treatment. A well-documented defense of leukemia cells is to shift the balance of the BCL-2 family in favor of the prosurvival proteins to protect against such intra- and extracellular stimuli. Small molecule inhibitors targeting the prosurvival proteins, named ‘BH3 mimetics’, have come to the fore in recent years to treat hematological malignancies, both as single agents and in combination with standard-of-care therapies. The most significant example of these is the BCL-2-specific inhibitor venetoclax, given in combination with standard-of-care therapies with great success in AML in clinical trials. As the number and variety of available BH3 mimetics increases, and investigations into applying these novel inhibitors to treat myeloid leukemias continue apace the need to evaluate where we currently stand in this rapidly expanding field is clear.

## Facts

- Dysregulation of prosurvival BCL-2 proteins is highly implicated in the oncogenesis, progression, and therapy-resistance of myeloid leukemias.
- BH3 mimetics inhibit prosurvival BCL-2 proteins and re-balance the apoptotic pathway.
- The BCL-2-specific BH3 mimetic venetoclax has had significant clinical success in acute myeloid leukemia in combination with standard therapy.
- Various BH3 mimetics in combination with standard-of-care therapies are currently under investigation in myeloid leukemias.

## Open Questions

- Could BH3 mimetics be useful in the treatment of chronic myeloid leukemia, either alone or in combination with tyrosine kinase inhibitors?
- Will the recent clinical success of the BCL-2 inhibitor venetoclax in combination with cytarabine or hypomethylating agents in acute myeloid leukemia encourage further combinatorial studies of venetoclax with other standard-of-care therapies in this disease?
- Is there scope for using MCL-1 and BCL-xL inhibitors in myeloid leukemias in a clinical setting?

## Introduction

Apoptosis is the best-described form of programmed cell death, discrete from other forms of cell death such as autophagy, necroptosis, ferroptosis, and pyroptosis^1,2^. An apoptotic cell displays morphological changes including nucleus shrinkage and membrane blebbing. Apoptotic cells undergo DNA degradation, cleavage of intracellular structures, and loss of mitochondrial function^1^. The term ‘apoptosis’ refers to two pathways distinct in initiation. The extrinsic apoptotic pathway is triggered via death receptor binding at the cellular membrane^3^, while the intrinsic, or ‘mitochondrial’, the pathway is regulated by B-cell leukemia/lymphoma-2 (BCL-2) family of proteins^4,5^. The two pathways crosstalk at the level of truncated BH3-interacting domain death agonist (tBID) activation, which occurs concurrently with the instigation of a caspase cascade in the context of the extrinsic pathway, and prior to activation of the multidomain proapoptotic effector proteins in the case of the intrinsic pathway.

During neoplastic transformation cells face numerous signals, including DNA damage, which would initiate apoptosis in healthy cells; however malignant cells hijack the apoptotic machinery to evade cell death^6^. One extensively studied example is an over-reliance on the BCL-2 family^7^. BCL-2 was first described in relation to survival from cell death due to its role as a driver of follicular lymphoma (FL)^8^. Other prosurvival members of the BCL-2 family have been implicated to varying degrees in the pathogenesis of other hematological malignancies including acute myeloid leukemia (AML)^9,10^, chronic myeloid leukemia (CML)^11–15^, multiple myeloma^16^, diffuse large B-cell lymphoma^17^, and acute lymphoblastic leukemia (ALL)^18^.

In recent years, the implications of BCL-2 family dependence in hematological malignancies has resulted in widespread and sustained effort to investigate whether this can be exploited to selectively eliminate cancerous cells.

### The intrinsic apoptotic pathway

**Fig. 1:**
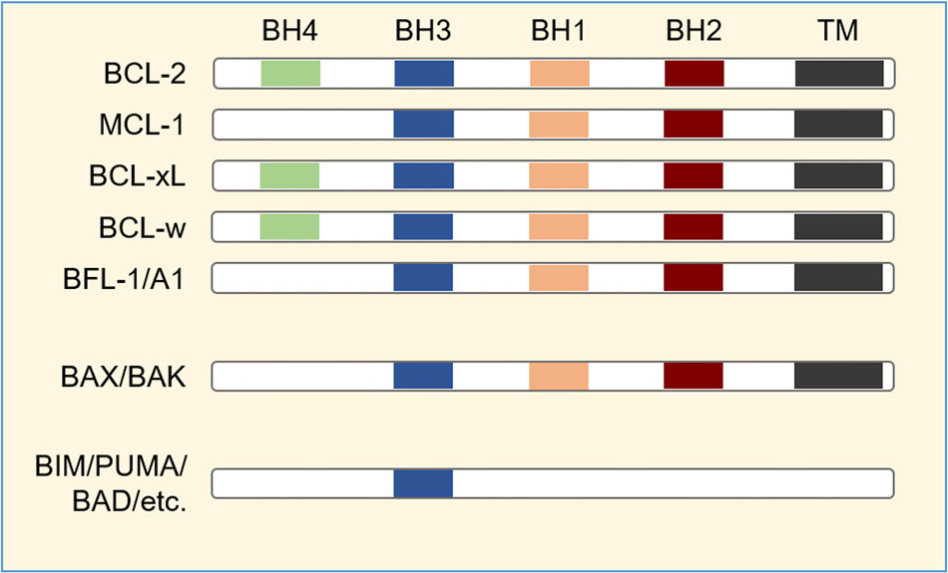
Members of the BCL-2 family of proteins share BCL-2 homology (BH) domains and are grouped according to function and structure. Figure not to scale. TM transmembrane domain.

Within the intrinsic apoptotic pathway, the decision to commit to cell death occurs at the mitochondrial outer membrane (MOM) and is dictated by a balance between opposing factions within the BCL-2 family^19^. The family can be divided primarily by function (prosurvival or proapoptotic), and latterly by structure (Fig. 1). BCL-2, along with myeloid cell leukemia-1 (MCL-1), B-cell lymphoma-extra large (BCL-xL; BCL2L1), B-cell lymphoma-w (BCL-w), and BCL-2-related gene expressed in fetal liver-1 (Bfl-1; A1), are able to inhibit apoptosis and contain three to four regions of conserved homology termed BCL-2 homology (BH) domains 1-4. Within the proapoptotic group of proteins there are two subgroups: (1) the multidomain proteins BCL-2-associated X protein (BAX) and BCL-2 homologous antagonist killer (BAK), and; (2) the BH3-only proteins, including BCL-2-interacting mediator of cell death (BIM), a p53-upregulated modulator of apoptosis (PUMA; BBC3), BCL-2 associated death promoter (BAD), NOXA (phorbol-12 myristate-13-acetate-induced protein 1; PMAIP1), BH3-interacting domain (BID), BCL-2-interacting killer (BIK), BCL-2-modifying factor (BMF) and Harakiri (HRK).

When activated, BAX and BAK oligomerize to form toroidal structures within the membrane^20^. The result is MOM permeabilisation (MOMP), the efflux of proteins from the mitochondrial intermembrane space and subsequent loss of membrane potential. Among the proteins released from the mitochondria through the BAX/BAK pores is cytochrome c, which combines with apoptotic protease-activating factor 1 (APAF1) and caspase-9 to form the apoptosome, a multi-protein complex that activates the effector caspases-3, -6 and -7^21^.

**Fig. 2:**
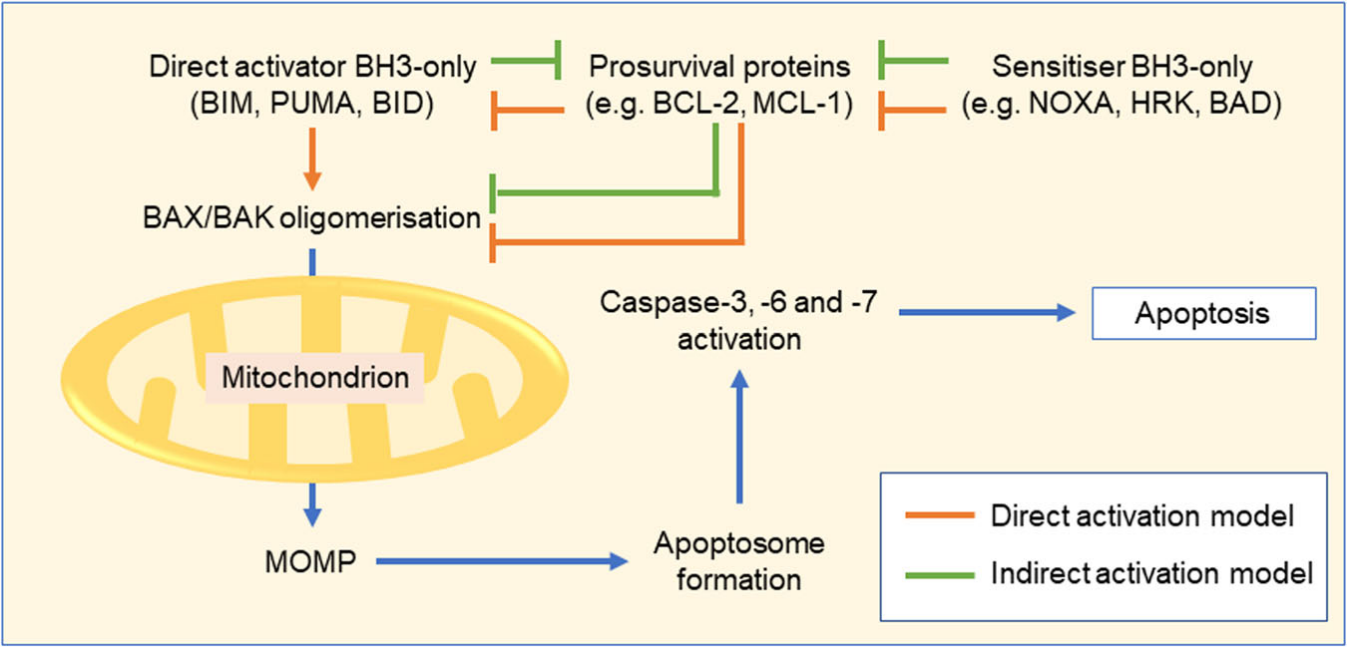
Execution of the intrinsic apoptotic pathway requires activation of BAX and BAK to form pores in the outer mitochondrial membrane resulting in mitochondrial outer membrane permeabilisation (MOMP). BAX/BAK activation occurs through either the direct activation model, indirect activation model or a balance of both.

The mechanism of BAX and BAK activation may occur via the direct activation and/or the indirect activation model (Fig. 2)^22^. The direct activation model suggests that BAX and BAK exist in an inactive conformation until activated by a subset of BH3-only proteins termed ‘direct activators’, including BIM, PUMA, and BID. These direct activators are sequestered by the prosurvival proteins and released upon inhibition of the latter by further ‘sensitiser’ BH3-only proteins (such as NOXA, HRK, and BAD). Conversely, the indirect activation model postulates that BAX/BAK are constitutively active and are inhibited by the prosurvival BCL-2 proteins; in response to a death stimulus, BH3-only proteins in turn inhibit the prosurvival proteins, thereby lifting the inhibition of BAX/BAK. In both models, the inhibition of the prosurvival proteins and subsequent release of either direct activator BH3-only proteins or BAX/BAK is the initiating step in triggering apoptosis^23^.

BH3-only proteins display differing binding affinities to the prosurvival proteins (e.g., NOXA binds with high specificity to MCL-1, while HRK binds exclusively with BCL-xL)^24^. Along with the evidence that malignant cells can evade apoptosis through over-reliance on the prosurvival BCL-2 proteins, this has led to the development of highly specific small molecule inhibitors of the prosurvival proteins. The inhibitors are rationally designed to mimic the BH3-only protein known to inhibit the prosurvival protein of interest and, as such, are termed ‘BH3 mimetics’^25^.

In this review, we focus on the use of BH3 mimetics within the myeloid leukemias, specifically CML and AML. We highlight the dependencies on these proteins, the compounds developed to take advantage of these discoveries and investigations conducted which combine BH3 mimetics with standard-of-care therapies, concluding with future directions for the field.

## Dysregulation of the intrinsic apoptotic pathway in CML

CML is typified by the Philadelphia chromosome, a (t(9;22)(q34;q11)) chromosomal translocation arising in a hematopoietic stem cell (HSC), leading to the expression of the fusion oncoprotein BCR-ABL^26,27^. This constitutively active tyrosine kinase sits at the epicenter of a complex signaling network that contributes to the malignant transformation of HSC into leukemic stem cells (LSC) which overpopulate the hematopoietic system with a myeloid bias^28^.

**Fig. 3:**
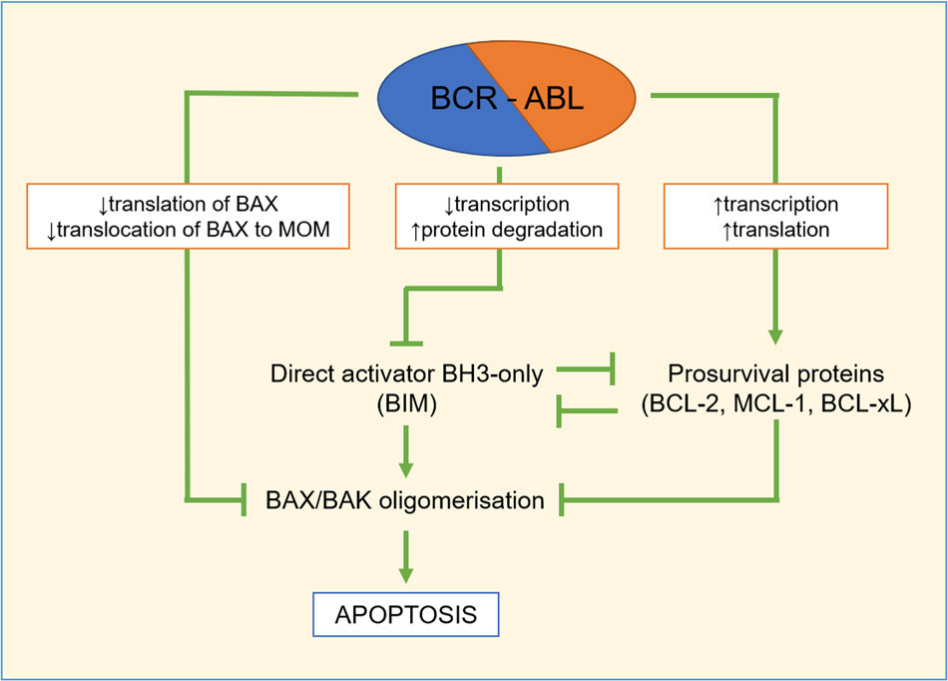
BCR-ABL affects the intrinsic apoptotic pathway through upregulation of prosurvival and downregulation of proapoptotic proteins. Targeting of these protein results in a shift away from apoptosisin CML cells. Black arrows indicate BCR-ABL-mediated up- or downregulation.

The current standard-of-care treatment for chronic phase (CP) CML is BCR-ABL-specific tyrosine kinase inhibitors (TKIs) such as imatinib (Gleevec®)^29^. For patients whose cancer becomes TKI-refractory, the disease may progress to blast phase (BP), with a 6 to 11-month survival rate and few treatment options available^30^. The BCL-2 family of proteins has been implicated in both CML development and progression; therefore, targeting the intrinsic apoptotic pathway may be a viable therapeutic option (Fig. 3).

### BIM in CML

Evasion of apoptosis in response to cytokine withdrawal is one of the most consistently observed effects of BCR-ABL; this withdrawal results in BIM upregulation in normal hematopoietic progenitors, but the effect is abolished in BCR-ABL-transformed cells^31^. BCR-ABL downregulates *BIM* transcription and labels the BIM protein for degradation through mitogen-activated protein kinase (MAPK) phosphorylation, with BIM levels restored by inhibiting BCR-ABL with TKIs, such as imatinib. Silencing of BIM effectively rescues CML cells from apoptosis caused by imatinib^31,32^. BCR-ABL therefore supports CML cell survival, at least in part, through the downregulation of BIM.

### Prosurvival BCL-2 proteins

*MCL-1* mRNA and MCL-1 protein are expressed constitutively in a BCR-ABL-dependent manner in CML regardless of disease stage^12^. Upregulation of BCL-xL has been observed in *BCR-ABL-*transformed HL-60 and BaF3 cells, while inhibition of the Akt/Protein kinase B pathway was found to reverse the upregulation of BCL-xL in the latter^33,34^. Investigations using apoptosis-resistant BCR-ABL^+^ mice suggest BCL-2 mutations in myeloid progenitors may be critical in the transition of BCR-ABL^+^ leukemias to advanced stage disease^13^. Further, inhibiting both BCL-2 and BCR-ABL is sufficient to induce apoptosis in CML stem cells in a murine CML model and TKI-resistant BP-CML patient samples^35^.

### Role of BAX

The serine/threonine-specific protein kinase Akt/Protein kinase B, a downstream target of BCR-ABL, is constitutively active in CP-CML and BP-CML cells; Akt inhibits a conformational change in BAX required for translocation to the mitochondrial membrane, thus hindering MOMP in response to cellular stress^36,37^. In CML cells expressing high levels of BCR-ABL, this movement of BAX is prevented^38^.

The microRNA miR-29b, able to increase the expression of BAX, is inhibited by BCR-ABL and is downregulated in BP-CML^39,40^. Overexpression of miR-29b in the CML cell line K562 has been shown to halt proliferation and induce apoptosis, indicating an important role for this miR in regulating cell death^39^.

Thus, CML cells can hijack BAX both at the translational and conformational levels, thereby decreasing sensitivity to cytotoxic stimuli and a further balance shift of the BCL-2 family proteins in favor of cell survival.

## Dysregulation of the intrinsic apoptotic pathway—AML

AML is the most common myeloid malignancy in adults, with an incidence rate of 3–5 cases per 100,000 per year and a median age of 68 years at diagnosis. AML covers a genetically heterogenous group of disorders of myelopoiesis with immature myeloid blasts in the bone marrow, blood, and extramedullary tissues. These blast cells out-compete normal hematopoiesis leading to the disease phenotype of fever, infection, anemia, bruising, and bleeding^41^.

Classification of AML is based on the World Health Organization and European Leukemia Network criteria, which rely on morphology, immunophenotyping, and the detection of underlying genetic lesions including both recurrent cytogenetic and molecular abnormalities^42^. Conventional karyotyping is the mainstay of risk stratification in AML and is complemented by fluorescence in situ hybridization analysis and RT-PCR for the targeted detection of specific recurrent genetic abnormalities^43^.

Next-generation sequencing can further stratify AML based on the presence or absence of cooperating mutations involved in driving the disease, encompassing epigenetic regulators, cell signaling and proliferation pathways, master hematopoietic transcription factors, and tumor suppressors^44^. The cytogenetic and molecular abnormalities present at diagnosis influence prognosis and clinical management and are used to subtype patients appropriately into favorable, intermediate, and adverse prognostic categories^41,42^.

This complex genomic landscape, combined with other co-morbidities and age at onset, makes treatment and management of AML patients challenging and, increasingly, an individualized approach is required. The therapeutic pathway taken will depend not only on the underlying genomic lesions, but also the age and fitness of the patient. Younger and fit older patients will receive high-intensity induction chemotherapy, followed by either consolidation chemotherapy or a stem cell transplant, dependent on response to therapy and genetic lesions present at diagnosis. Until recently, patients deemed unfit to tolerate intensive chemotherapy would receive either low dose cytarabine (LDAC), hypomethylating agents (HMA), or palliative treatment such as hydroxyurea in addition to supportive care^41^. LDAC and HMA may achieve remissions in a minority of patients, but are not curative and almost all patients will relapse. Small molecule inhibitors may also be included for patients with specific genetic lesions, e.g. midostaurin for patients with a fms-like tyrosine kinase 3 (FLT3) mutation^45^.

### Role of BCL-2 family in AML

The journey from the identification of BCL-2 dependence in AML to the successful development and clinical application of the BCL-2-specific inhibitor venetoclax is a triumph of modern cancer therapy. BCL-2 was found to be expressed in AML CD34^+^ progenitor cells and promyelocytes while this expression was absent in their heathy counterparts, and evidence was presented that induction chemotherapy resulted in selection for leukemic CD34^+^ cells expressing high levels of BCL-2^46^. Later, it was shown that BCL-2 is essential for the maintenance of cancer cells in a murine model of leukemia, in the first example of the functional removal of a BCL-2 family prosurvival protein resulting in cancer regression^47^.

BCL-2 expression has also been shown to be significantly upregulated in newly diagnosed AML patients (range of 34–87%) and relapsed AML patients^10,48–50^. Patients with elevated BCL-2 tend to present with higher percentage of peripheral blasts^48^, with over-expression also correlating with CD34 and CD117 positivity and poorer response to chemotherapy^10,49,50^, suggesting a more primitive phenotype.

An early investigation saw the application of a cell-permeable BCL-2 binding peptide, based on the structure of BAD, in HL-60 cells in vitro and human myeloid leukemia cells in a murine model, resulting in leukemic cell death^51^. This was followed swiftly by the description of HA14-1, a small molecule compound able to bind to the BCL-2 surface pocket and capable of inducing caspase-dependent apoptosis in HL-60 cells^52^.

Other BCL-2 family proteins, including BCL-xL and MCL-1 have been implicated in the pathogenesis of AML^53–55^. BCL-xL and BAD, along with BCL-2, are upregulated in the majority of AML stem/progenitor cell populations, compared to normal hematopoietic stem/progenitor cells (HSPCs), with induction chemotherapy resulting in a further upregulation of BCL-2 and BCL-xL^46,54^. MCL-1 is consistently high in the majority of newly diagnosed AML patients and has been associated with relapse^56,57^. MCL-1 is also linked to stem cell survival, especially in FLT3-internal tandem duplication (FLT3-ITD) AML stem cells^58^.

A critical role of MCL-1 in cell survival was demonstrated in an elegant study using bone marrow HSCs/HSPCs transformed with the oncogenes mixed-lineage leukemia (MLL)-eleven nineteen leukemia (MLL-ENL) and MLL-ALL1-fused gene from chromosome 9 (MLL-AF9), and corresponding AML mouse models. Depletion of Mcl-1 led to the death of cells in vitro and reduced disease burden in AML-afflicted mice, with cell death being rescued by overexpressing Bcl-2 or Mcl-1^57^.

Due to the heterogeneity of AML, studies indicate that cells may be ‘addicted’ to BCL-2, MCL-1, or both depending on the genomic landscape of the patient at diagnosis^59^. If BH3 mimetics are to be used successfully clinically in the management of AML, patient-specific prediction of BCL-2 family dependency, potentially by BH3 profiling may well be essential^60,61^.

## The rise of BH3 mimetics

The developmental journey of BH3 mimetics to clinical use has been extensively covered, including the excellent reviews by Lessene et al.^62^ and Leverson et al.^63^. One of the first BH3 mimetics developed, following HA14-1, was ABT-737, a small molecule with high binding affinity to BCL-2, BCL-xL, and BCL-w^16,64^. ABT-737 represents the first example of an anti-cancer drug designed specifically to target a protein-protein interaction, and was identified through the structure-activity relationships (SAR) by nuclear magnetic resonance (NMR) method and site-directed parallel synthesis, a triumph of modern, rational cancer therapy design^64^.

The major limitation of ABT-737 was the lack of oral bioavailability, prompting the development of ABT-263 (navitoclax)^65^. Navitoclax is a dual inhibitor of BCL-2/BCL-xL, and its application as a monotherapy in relapsed/refractory (R/R) CLL was promising, with a 35% partial response rate, though 28% of patients experienced grade 3/4 thrombocytopenia due to the requirement of BCL-xL in the development of platelets^66,67^. The serious adverse effects associated with BCL-xL inhibition in vivo was addressed with the development of ABT-199 (venetoclax, Venclexta®), a highly specific BCL-2 inhibitor that induced less thrombocytopenia^68^.

Venetoclax was first described in 2013^68^, and since has been approved in the US for the treatment of CLL with 17p deletion (2016), in combination with rituximab (Rituxan®) for previously untreated CLL (2018), newly diagnosed AML in combination with HMA or LDAC where intensive induction chemotherapy is not possible (accelerated approval in 2018, full approval in 2020) and in combination with non-chemotherapeutics for previously untreated CLL (2019). The path to these approvals in AML will be addressed further in this review.

Of interest in the context of the current COVID-19 pandemic, NHS England granted temporary emergency approval of venetoclax in specific AML patient groups. Venetoclax treatment can be delivered on an outpatient basis, allowing for reduced attendance at the clinic for the duration of the pandemic until regular treatment can be resumed.

Resistance to venetoclax can occur through upregulation of other BCL-2 prosurvival proteins, and subsequent targeting of these proteins with alternative BH3 mimetics or inhibiting upstream regulatory pathways is often effective in overcoming resistance^69–71^. To this end, and especially when targeting cancers in which BCL-2 is not the primary prosurvival BCL-2 protein, other BH3 mimetics may come to the fore of clinical studies in the future (Table 1).

**Table 1:** Currently commercially available BH3 mimetics and related compounds are known to have been investigated in the context of hematological malignancies, with those in current and/or previous clinical trials for leukemias denoted with an asterisk (*).

Compound	Target	Published	PubMed ID
ABT-737	BCL-2, BCL-xL, BCL-w	2005	15902208
ABT-263 (navitoclax)*	BCL-2, BCL-xL, BCl-w	2008	18451170
ABT-199 (venetoclax)*	BCL-2	2013	23291630
GX15-070 (obatoclax)*	Pan-BCL-2	2005	16304385
WEHI-539	BCL-xL	2013	23603658
S1	Pan-BCL-2	2011	20503275
Apogossypolone (ApoG2)	Pan-BCL-2	2008	18769131
BI97C1 (sabutoclax)	BCL-2, BCL-xL, A1, MCL-1	2010	20443627
TW-37	BCL-2, BCL-xL, MCL-1	2006	16951185
BXI-61, BXI-72	BCL-xL	2013	23824742
JY-1-106	BCL-xL, MCL-1	2013	23680104
MIM1	MCL-1	2012	22999885
UMI-77	MCL1	2014	24019208
Marinopyrrole A (maritoclax)	MCL-1	2012	22311987
A-1331852 and A1155463	BCL-xL	2015	25787766
A1210477	MCL-1	2015	25590800
S63845	MCL-1	2016	27760111
S55746 (BCL201)*	BCL-2	2018	29732004
ML311 (EU-5346)	MCL-1, A1	2012	23762927
HA14-1	BCL-2	2000	10860979
2-Methoxy antimycin A3	BCL-2, BCL-xL	2001	11175751
AMG176*	MCL-1	2018	30254093
Gossypol (AT101)*	Pan-BCL-2	2003	13678404
AZD5991*	MCL-1	2018	30559424
S64315 (MIK665)*	MCL-1	Unpublished	N/A
A385358	BCL-xL	2006	16951189
VU0661013	MCL-1	2018	30185627
ML311	MCL-1	2013	23762927
AZD4320	BCL-2, BCL-xL	2019	29931583

## BH3 mimetics with standard-of-care therapies

**Fig. 4:**
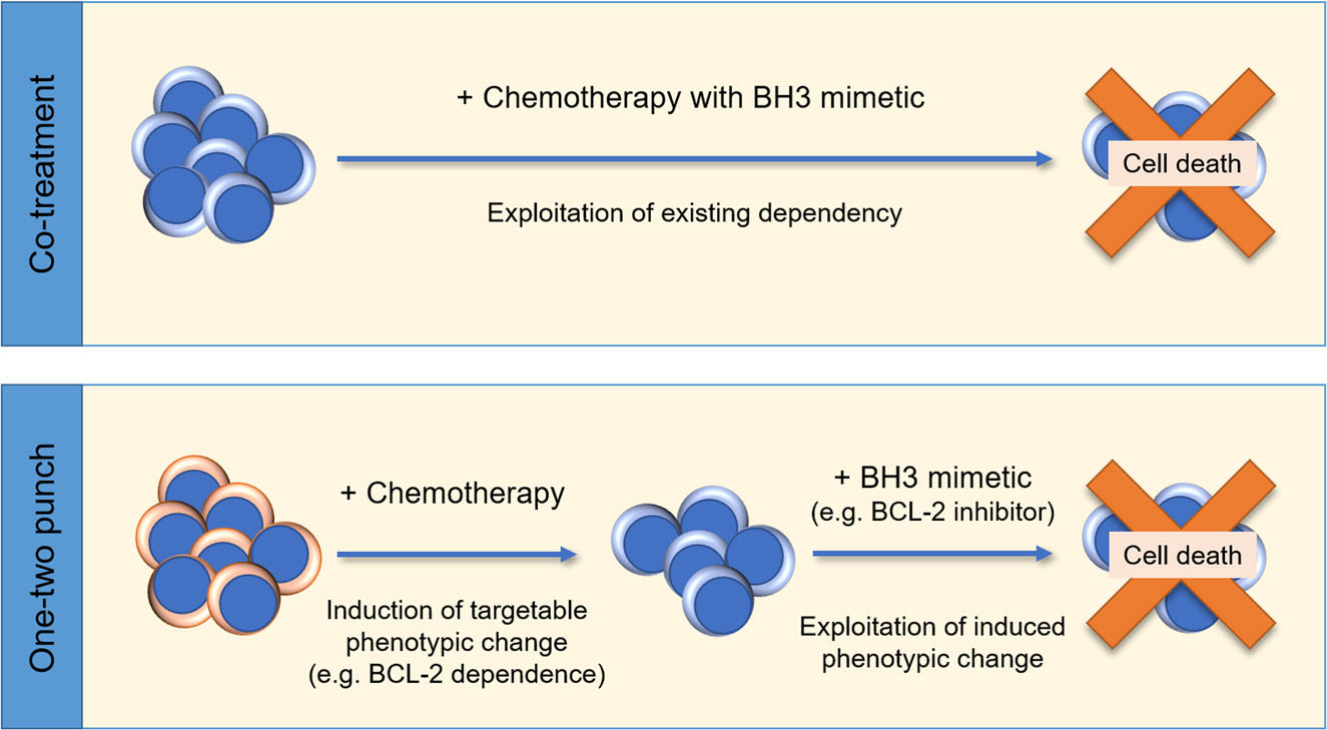
Differing treatment methods deserve consideration for the application of BH3 mimetics in combination with standard therapies. Proposed methods for combination treatments with BH3 mimetics include co-treatment with a chemotherapy or inducing an increase in dependency on a BCL-2 prosurvival protein by treatment with chemotherapy which can then be targeted with a BH3 mimetic (one-two punch method).

Two methods of administering BH3 mimetics deserve consideration: co-treatment and the one-two punch method (Fig. 4). In the first, a BH3 mimetic is chosen based on the known BCL-2 dependency of the cancer cells to prohibit this defense against the chemotherapy of choice. The second method involves inducing a targetable change in the cancer cells. Treatment with the chemotherapy is used to induce cell death signaling in the cells, thereby making the cells more reliant on one or more of the BCL-2 family proteins; the cells can then be targeted with a selected BH3 mimetic. To be most effective, this may require several rounds of sequential treatment with chemotherapy, alternating with a BH3 mimetic. In both cases, dependency can be measured through ex vivo mimetic treatment or methods such as BH3 profiling^72^. This approach may be particularly valuable in diseases such as AML where complex genetic landscapes make it more challenging to predict individual patient response to treatment.

Although a vast array of highly specific BH3 mimetics targeting different members of the BCL-2 family are available to researchers at the bench, venetoclax is the only one in common use clinically. This is due to the adverse effects of targeting BCL-xL and MCL-1, namely thrombocytopenia^66,73^ and potentially cardiac toxicity^74,75^, respectively. If BH3 mimetics targeting other family members are to be used clinically in the future, fine-tuning to improve tolerability will be required. Drug dosages that induce cancer cell death must be lower than those which damage healthy tissue; it is here that the one-two punch method could be utilized, to heighten the sensitivity of the cancer cells to the mimetic, and to allow recovery of normal tissue in between rounds of mimetic treatment.

### Chronic myeloid leukemia

One of the biggest challenges in treating CP-CML is TKI-resistance. Among other pathways, overexpression of the BCL-2 prosurvival proteins and low levels of the proapoptotic BIM protein have been linked to TKI-resistance, leading to investigations into combining TKIs with BH3 mimetics. The persistence of CML stem cells also represents a barrier to the successful elimination of the disease;^28^ these LSCs are resistant to TKI treatment, with alternative methods for eradicating this population therefore required^76,77^.

### Pre-clinical combinations of TKIs with BH3 mimetics in CML

In terms of circumventing TKI-resistance via BCL-2 family imbalance mechanisms, co-treatment using TKI with a BH3 mimetic has shown efficacy. ABT-737 re-sensitized the CML cell line K562 to imatinib-induced cell killing in cells with imatinib-resistance mediated by BIM knockdown or BCL-2 overexpression^32^. This effect was also observed in *Bim*^*−/−*^*Bad*^*−/−*^ *BCR-ABL*-transformed murine fetal liver-derived myeloid progenitor cells. These findings demonstrate that imatinib-resistance resulting from alterations in the BCL-2 family can be overcome through co-treatment with a BH3 mimetic

Analysis of CP-CML East Asian patients found a *BIM* deletion polymorphism, resulting in expression of BIM lacking the BH3 domain, and was linked to TKI-resistance^78^. Crucially, although TKI-resistance is usually associated with BCR-ABL kinase domain mutations^79^, it was found that patients with the polymorphism were less likely to have a BCR-ABL kinase domain mutation, suggesting an almost mutually exclusive mechanism of TKI-resistance and that treating these patients with further TKIs may be of little advantage. Co-treatment with ABT-737, however, restored imatinib-induced cell death in BIM-mutated CML cell lines and patient samples with the polymorphism.

More recently, BH3 mimetics in combination with TKIs have been used to target the CML progenitor compartment, with notable success in a number of CML disease models^35,80–82^. These include reducing the colony-forming capacity of CP-CML progenitors (CD34^+^ CP-CML cells, venetoclax with imatinib)^80^, reducing leukemic burden and long-term engraftment potential and increasing overall survival in CML murine models (*Bcr-Abl1*^*+*^ Tet-off Lin^-^Sca-1^+^cKit^+^ cells, venetoclax with nilotinib)^35^, and increasing apoptosis in BP-CML progenitors (CD34^+^CD38^−^ BP-CML cells, venetoclax with nilotinib)^35^, (CD34^+^ BP-CML cells, ABT-737 with imatinib)^81^ and CP-CML progenitors (CD34^+^CD38^-^ CP-CML cells, ABT-737 with imatinib/nilotinib)^82^.

In summary, for TKI-resistance mediated by the proapoptotic or prosurvival proteins, BH3 mimetics appear effective in redressing the balance, and re-sensitizing CML cells to TKIs.

However, due to the effectiveness of TKIs alone, there is very little clinical trial activity investigating combinations of BH3 mimetics with TKIs. One phase 2 clinical trial is currently recruiting, combining venetoclax with the TKI dasatinib in early CP-CML, with the primary endpoint of assessing the proportion of patients achieving major molecular response after 12 months of therapy (NCT02689440)^83^. To date, no results are available for this trial. A second phase 2 study combining decitabine, ponatinib and venetoclax in blast phase CML is also underway, with a primary endpoint of overall response rate (NCT04188405)^84^. To date, there are no clinical trials in CML of MCL-1 or BCL-xL inhibitors.

### Acute myeloid leukemia

**Fig. 5:**
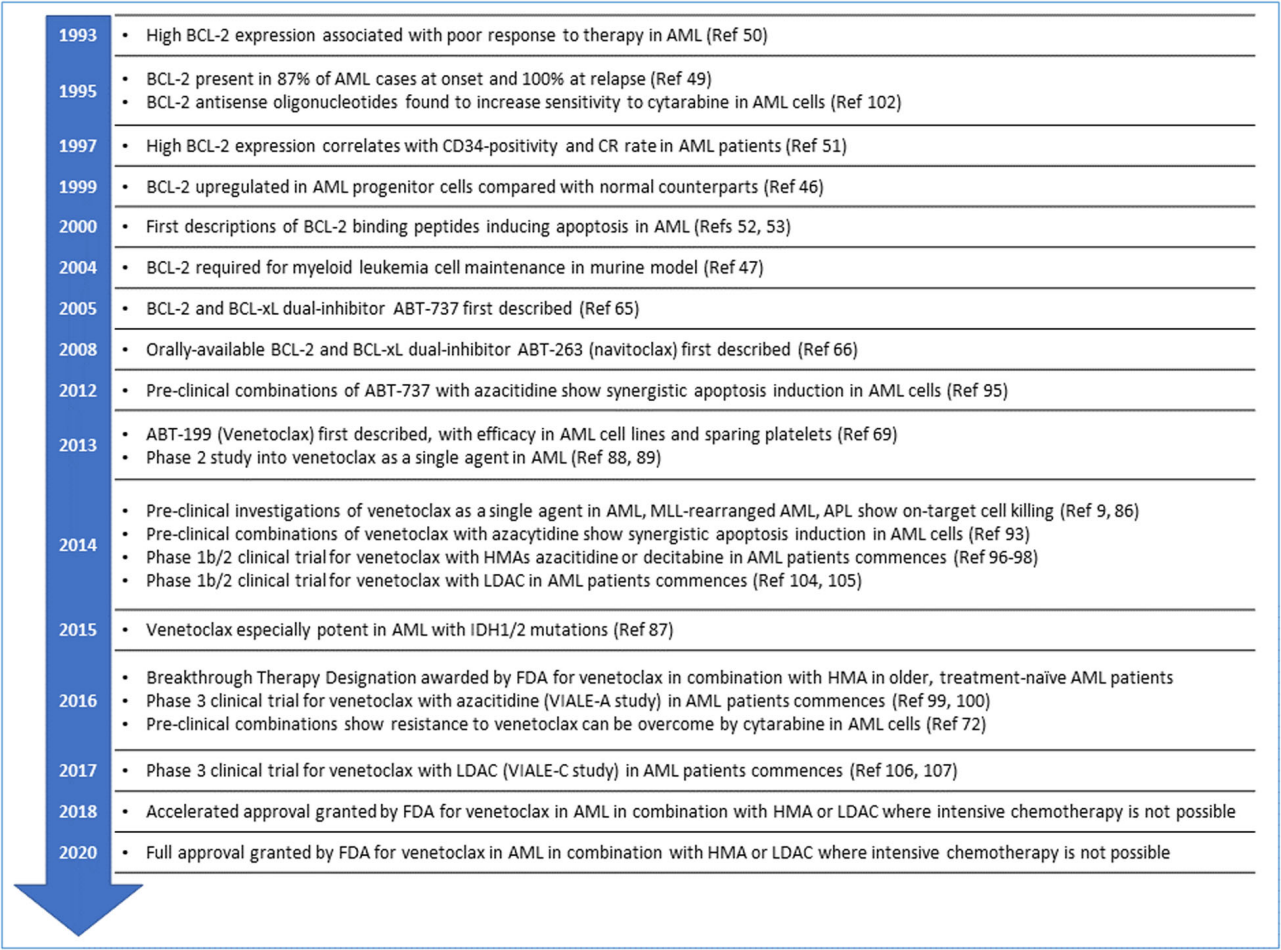
The journey from development to clinical application of venetoclax is a triumph of rational cancer therapy design. Extensive experimental data identifying the prosurvival BCL-2 family members as potential therapeutic targets, focused preclinical work combining BH3 mimetics with standard-of-care therapies in AML, and highly promising, international clinical trials have led to FDA approval of venetoclax with HMA or LDAC in an impressively short timeframe.

The success story of venetoclax in AML is one that cannot be understated, especially for the exceptionally short timeframe from the first description of venetoclax in 2013^68^ to full approval by the FDA for venetoclax plus HMA or LDAC in older, unfit AML patients in 2020. This speaks to the substantial and convincing work into the BCL-2 family in AML, through pre-clinical studies of venetoclax alone and in combination with other therapies, to the large international trials that resulted in directly improving patient care. Figure 5 illustrates this remarkable path.

Along with mounting evidence for the role of BCL-2 in AML cell survival, early preclinical studies into venetoclax as a monotherapy in AML cell lines, patient samples, and a murine xenograft model demonstrated on-target cell killing^9^, with particular sensitivity to venetoclax seen in AML cells harboring the MLL fusion genes and in acute promyelocytic leukemia (APL) cells^85^. Interestingly, venetoclax is especially potent in AML cells with isocitrate dehydrogenase 1 and 2 (IDH1/2) gene mutations; these proteins have been implicated in increasing BCL-2 dependence^86^. In a phase 2 clinical trial in relapsed/refractory AML, single-agent venetoclax had an overall response rate of 19%, while 33% (4 out of 12) of patients with IDH1/2 mutations demonstrated CR^87,88^.

There is also increasing use and success with the MCL-1 inhibitors AMG-176, AMG-397, and S64315 in pre-clinical models of AML, regardless of the presence of specific genetic lesions^59,89^.

Despite the limited clinical success of venetoclax as a monotherapy in AML, evidence supporting the combination of venetoclax with standard-of-care therapies in AML is encouraging. For example, treatment of AML patients with cytarabine and idarubicin has been shown to increase BCL-2 expression in the CD34^+^ compartment^46^ and high de novo expression of BCL-2 is correlated with poor response to treatment^90,91^, indicating scope for venetoclax combinations in AML.

### Azacitidine with BH3 mimetics in AML

Pre-clinical synergistic cytotoxic effects were shown by several groups when combining ABT-737 or venetoclax with the HMA azacitidine in AML cell lines and primary patient samples in vitro^92–94^. RNA-interference screening identified the proapoptotic BCL-2 proteins as potential targets for enhancing the effects of azacitidine^92^, and later it was shown that ABT-737 was a more potent agent than venetoclax when used in combination with azacitidine, due to the variable expression of the prosurvival BCL-2 proteins between patients^93^.

These promising findings led to the development of clinical trials investigating a BCL-2-targeting BH3 mimetic in combination with azacitidine in myeloid leukemias. A phase 1b trial comparing the combination of venetoclax with azacitidine or decitabine in AML patients over 65 years of age with treatment-naïve AML, and who were ineligible for intensive chemotherapy, demonstrated an extremely promising 73% CR or CR with incomplete count recovery (CRi) for the cohort receiving HMA and 400 mg venetoclax^95–97^. These results led, in 2016, to the FDA granting venetoclax Breakthrough Therapy Designation in combination with HMA in older patients with treatment-naive AML.

The large VIALE-A phase 3 trial that followed combined azacitidine with 400 mg venetoclax and compared against an azacitidine plus placebo control group, enrolling 443 untreated AML patients who were either over the age of 75 or could not tolerate standard chemotherapy, or both^98^. At an interim analysis, overall survival (OS) and CR were increased from the control (OS: 9.6 months; CR: 28.3%) to the azacitidine plus venetoclax group (OS: 14.7 months; CR: 66.4%)^99^. By 2018, the FDA had granted accelerated approval for venetoclax in combination with HMA in patients who cannot receive induction chemotherapy, and full approval granted for this indication in 2020.

### Cytarabine with BH3 mimetics in AML

Incorporation of cytarabine into the DNA of rapidly dividing cells induces cell cycle arrest in the S phase through inhibition of DNA synthesis^100^. Pre-clinical inhibition of BCL-2 by antisense oligonucleotides, obatoclax or venetoclax in combination with cytarabine has been shown to significantly enhance cell death in AML cell lines and patient samples^71,101,102^. Cytarabine-mediated reduction of MCL-1 expression may also contribute to the synergistic action of BCL-2 and/or BCL-xL inhibition in these cells^71^.

In phase 1b/2 clinical trials with patients over the age of 65, the combination of venetoclax with LDAC had a CR rate of 54% and OS of 10.1 months, though these rates were increased to 62% and 13.5 months respectively for patients with no prior HMA treatment^103,104^.

As with the azacitidine plus venetoclax success, a large, international phase 3 clinical trial, VIALE-C, was quick to follow^105^. Enrolled participants were ineligible for intensive chemotherapy and were treated either with venetoclax or placebo with LDAC. As of summer 2020, OS was 4.1 and 7.2 months and CR rates were 13% and 48% in the control and venetoclax arms, respectively^106^. This trial continues in follow-up, but in 2020 these promising initial results led to FDA full approval of LDAC with venetoclax in treatment-naive AML patients.

### Further pre-clinical combinations of conventional therapy with BH3 mimetics in AML

The success of venetoclax in AML in combination with the standard-of-care therapies HMA and LDAC has led to investigations into combining this BH3 mimetic with other clinically available therapies. Here we will briefly describe some of these pre-clinical investigations.

#### Midostaurin

The apoptotic response to the FLT3 kinase inhibitor midostaurin in FLT3-ITD-positive primary AML samples and cell lines is enhanced in the presence of venetoclax^107^. FLT3-ITD upregulates MCL-1 through STAT5 activation and the Akt pathway; therefore, inhibition of FLT3-ITD and treatment with venetoclax concomitantly removes the protection of both MCL-1 and BCL-2, rendering the cell sensitive to apoptosis^58,108^.

#### Sorafenib

Sorafenib is a multi-kinase inhibitor targeting RAF, PDGFRB, VEGFR2, FLT3, and KIT, and induces apoptosis in AML cells via BIM and downregulation of MCL-1^109,110^. Further sensitizing cells to BIM with BH3 mimetics potentiates the apoptotic effect of sorafenib, as seen in combination with obatoclax and venetoclax^111^.

#### All-trans retinoic acid (ATRA)

MCL-1 overexpression impedes the ability of ATRA to induce growth arrest and differentiation in APL and combining ATRA with an MCL-1-interfering BH3 mimetic has been postulated to induce a greater cytotoxic response than ATRA alone^112^. However, the combination of JY-1-106 with ATRA was shown in one study to have little effect on reducing cell proliferation in HL-60, an APL cell line^113^.

#### Daunorubicin

Daunorubicin is a DNA-intercalating chemotherapeutic able to induce sphingomyelin hydrolysis and ceramide generation^114^. Overexpression of BCL-2 has been shown to prevent daunorubicin-induced apoptosis in AML cell lines through inhibition of X-linked inhibitor of apoptosis protein (XIAP) and degradation of Akt^115,116^. Removal of this BCL-2-mediated protection against daunorubicin has been shown to be effective at synergistically inducing apoptosis and growth inhibition in cell lines and in patient samples, using either ABT-737 or venetoclax^71,117^.

#### Combining mimetics

The possibility of combining BH3 mimetics with different target specificities is also under scrutiny in both CML and AML studies, although toxicity concerns have potentially held back investigations of this nature^70,118,119^. Combining BH3 mimetics has the advantage of disabling the cell’s ability to ‘switch’ between prosurvival proteins, a commonly reported resistance mechanism to BCL-2 inhibition, and thus overcoming the redundancy in the BCL-2 family system^70,120–122^.

### Further clinical trials with BH3 mimetics in AML

In contrast to the clinical success of venetoclax, clinical trials of MCL-1 inhibitors have been more problematic. Initially, there were difficulties in developing MCL-1 inhibitors as the binding site is shallower and less flexible than that of BCL-2 or BCL-xL^123^. Recently, however, 4 agents (S64315^124^, AMG176^125^, AMG397^126^, and AZD5991^127^) with activity against MCL-1 entered phase 1 clinical trials as single agents in AML (NCT02979366^128^, NCT02675452^129^, NCT03465540^130^, NCT03218683^131^, with a view to combining with venetoclax (NCT03672695 ^132^, NCT03797261^133^, NCT03218683^131^) or azacitidine (NCT02675452^129^), once dose-finding studies are completed. Importantly, CDK9 inhibitors (e.g., alvocidib, dinaciclib, CYC065, and AZD4573^134–137^) indirectly inhibit MCL-1. These agents have preclinical activity in AML, and a number of early phase clinical trials are ongoing. It will be important to determine if they have efficacy with a favorable safety profile.

The BCL-2/BCL-xL inhibitor navitoclax has undergone extensive clinical trial evaluation in solid tumor, lymphoid malignancies and myeloproliferative neoplasms, but not AML. Further development has been limited by the predicted and on-target side effect of thrombocytopenia^67^.

## Measuring BCL-2 family dependence

Heterogenous responses to BH3 mimetics occurs in patients, indicating a need for personalizing treatment approaches when considering these drugs. BH3 profiling, a technique developed to predict relative dependency on BCL-2, MCL-1, and BCL-xL, has been shown to be useful in predicting responses of patients with AML after treatment with venetoclax, as well as highlighting potential resistance mechanisms^88^.

The success of BH3 profiling in this regard has led to the incorporation of this technique in several clinical trials as a prognostic marker and determinant of response, most notably for myelodysplastic syndrome and AML^138–141^, but also in trials focusing on CLL^142^ and ALL^143^. Further, combining BH3 profiling results with basal expression data for the prosurvival BCL-2 proteins (termed ‘mitochondrial profiling’) has also been shown to be effective in indicating BCL-2 dependence^144^. In the case of the clinical trial NCT02520011^145^, demonstrable MCL-1 dependence in AML, as determined by mitochondrial profiling, was used to identify eligible patients, although this trial was later terminated due to slow enrollment.

In addition, protein and gene expression profiles of the target BCL-2 family members^146,147^ and expression of BH3-only proteins such as BIM^148^ have been shown to correlate with response to BH3 mimetics.

BH3 and mitochondrial profiling, along with gene and protein expression data, represent high-throughput methods with a fast turnaround time, often requiring few cells and with limited ex vivo exposure of patient samples. As these techniques are refined, there is a trend in the literature towards assays with high specificity and sensitivity for identifying patients who may benefit from BH3 mimetic treatment, warranting investigations into the point-of-care applications of these assays.

## Future directions

With substantial progress being made in the field of BCL-2-targeted therapies and our increasing understanding of dysregulation of this family in the myeloid leukemias, great strides have been made in bringing these areas together, as highlighted in this review.

A number of unanswered questions and areas for further investigation remain to be addressed. With the difficulties in targeting MCL-1 and BCL-xL, the identification of a therapeutic window is required, which could be addressed through sequential or alternating treatment strategies to allow time for healthy tissue recovery, or through potent combinations that would allow for a substantial reduction in the concentrations of BH3 mimetic required. Further, measuring BCL-2 family dependency in a point-of-care setting to refine treatment deserves additional scrutiny to determine clinical utility.

As clinical trials advance and standard treatment regimens incorporate BH3 mimetics to a greater degree, these novel therapeutic combinations may represent a significant step in the direction of targeted, personalized therapy for patients with myeloid leukemias.

## References

[1] Kerr JFR, Wyllie AH, Currie AR (1972). Apoptosis: a basic biological phenomenon with wide-ranging implications in tissue kinetics. Br. J. Cancer.

[2] Kroemer G (2009). Classification of cell death: recommendations of the Nomenclature Committee on Cell Death 2009. Cell Death Differ..

[3] Ashkenazi A (2015). Targeting the extrinsic apoptotic pathway in cancer: lessons learned and future directions. J. Clin. Invest..

[4] Vaux DL, Weissman IL, Kim SK (1992). Prevention of programmed cell death in *Caenorhabditis elegans* by human BCL-2. Science.

[5] Oberst A, Bender C, Green DR (2008). Living with death: the evolution of the mitochondrial pathway of apoptosis in animals. Cell Death Differ..

[6] Hanahan D, Weinberg RA (2000). The hallmarks of cancer. Cell.

[7] Singh R, Letai A, Sarosiek K (2019). Regulation of apoptosis in health and disease: the balancing act of BCL-2 family proteins. Nat. Rev. Mol. Cell Biol..

[8] Tsujimoto Y, Cossman J, Jaffe E, Croce CM (1985). Involvement of the BCL-2 gene in human follicular lymphoma. Science.

[9] Pan R (2014). Selective BCL-2 inhibition by ABT-199 causes on-target cell death in acute myeloid leukemia. Cancer Discov..

[10] Campos L (1993). High expression of BCL-2 protein in acute myeloid leukemia cells is associated with poor response to chemotherapy. Blood.

[11] Aichberger KJ (2005). Low-level expression of proapoptotic BCL-2-interacting mediator in leukemic cells in patients with chronic myeloid leukemia: role of BCR/ABL, characterization of underlying signaling pathways, and reexpression by novel pharmacologic compounds. Cancer Res..

[12] Aichberger KJ (2005). Identification of MCL-1 as a BCR/ABL-dependent target in chronic myeloid leukemia (CML): evidence for cooperative antileukemic effects of imatinib and MCL-1 antisense oligonucleotides. Blood.

[13] Jaiswal S (2003). Expression of BCR/ABL and BCL-2 in myeloid progenitors leads to myeloid leukemias. Proc. Natl Acad. Sci. USA.

[14] Handa H (1997). Bcl-2 and c-myc expression, cell cycle kinetics and apoptosis during the progression of chronic myelogenous leukemia from diagnosis to blastic phase. Leuk. Res..

[15] Sánchez-García I, Grütz G (1995). Tumorigenic activity of the BCR-ABL oncogenes is mediated by BCL2. Proc. Natl Acad. Sci. USA.

[16] Kline MP (2007). ABT-737, an inhibitor of BCL-2 family proteins, is a potent inducer of apoptosis in multiple myeloma cells. Leukemia.

[17] Shivakumar L, Armitage JO (2006). BCL-2 gene expression as a predictor of outcome in diffuse large B-cell lymphoma. Clin. Lymphoma Myeloma.

[18] Coustan-Smith E (1996). Clinical relevance of BCL-2 overexpression in childhood acute lymphoblastic leukemia. Blood.

[19] Youle RJ, Strasser A (2008). The BCL-2 protein family: opposing activities that mediate cell death. Nat. Rev. Mol. Cell Biol..

[20] Westphal D, Dewson G, Czabotar PE, Kluck RM (2011). Molecular biology of Bax and Bak activation and action. Biochim Biophys. Acta - Mol. Cell Res..

[21] Riedl SJ, Salvesen GS (2007). The apoptosome: signalling platform of cell death. Nat. Rev. Mol. Cell Biol..

[22] Letai A (2002). Distinct BH3 domains either sensitize or activate mitochondrial apoptosis, serving as prototype cancer therapeutics. Cancer Cell..

[23] Bock FJ, Tait SWG (2020). Mitochondria as multifaceted regulators of cell death. Nat. Rev. Mol. Cell Biol..

[24] Chen L (2005). Differential targeting of prosurvival Bcl-2 proteins by their BH3-only ligands allows complementary apoptotic function. Mol. Cell.

[25] Delbridge ARD, Strasser A (2015). The BCL-2 protein family, BH3-mimetics and cancer therapy. Cell Death Differ..

[26] Shtivelman E, Lifshitz B, Gale RP, Canaani E (1985). Fused transcript of abl and bcr genes in chronic myelogenous leukaemia. Nature.

[27] Ben-Neriah Y, Daley GQ, Mes-Masson A-M, Witte ON, Baltimore D (1986). The chronic myelogenous leukemia-specific P210 protein is the product of the bcr-abl hybrid gene. Science.

[28] Holyoake T, Jiang X, Eaves C, Eaves A (1999). Isolation of a highly quiescent subpopulation of primitive leukemic cells in chronic myeloid leukemia. Blood.

[29] Deininger MWN, Goldman JM, Lydon N, Melo JV (1997). The tyrosine kinase inhibitor CGP57148B selectively inhibits the growth of BCR-ABL-positive cells. Blood.

[30] Hehlmann R (2012). How I treat CML blast crisis. Blood.

[31] Kuribara R (2004). Roles of Bim in apoptosis of normal and Bcr-Abl-expressing hematopoietic progenitors. Mol. Cell Biol..

[32] Kuroda J (2006). Bim and Bad mediate imatinib-induced killing of Bcr/Abl+ leukemic cells, and resistance due to their loss is overcome by a BH3 mimetic. Proc. Natl Acad. Sci. USA.

[33] Amarante-Mendes G (1998). Bcr-Abl exerts its antiapoptotic effect against diverse apoptotic stimuli through blockage of mitochondrial release of cytochrome C and activation of caspase-3. Blood.

[34] Gesbert F, Griffin J (2000). Bcr/Abl activates transcription of the Bcl-X gene through STAT5. Blood.

[35] Carter BZ (2016). Combined targeting of BCL-2 and BCR-ABL tyrosine kinase eradicates chronic myeloid leukemia stem cells. Sci. Transl. Med..

[36] Kawauchi K, Ogasawara T, Yasuyama M, Ohkawa SI (2003). Involvement of Akt kinase in the action of STI571 on chronic myelogenous leukemia cells. Blood Cells, Mol. Dis..

[37] Yamaguchi H, Wang HG (2001). The protein kinase PKB/Akt regulates cell survival and apoptosis by inhibiting Bax conformational change. Oncogene.

[38] Keeshan K, Cotter T, McKenna S (2002). High Bcr-Abl expression prevents the translocation of Bax and Bad to the mitochondrion. Leukemia.

[39] Li Y (2013). MiR-29b suppresses CML cell proliferation and induces apoptosis via regulation of BCR/ABL1 protein. Exp. Cell Res..

[40] Machová Polaková K (2011). Expression patterns of microRNAs associated with CML phases and their disease related targets. Mol. Cancer.

[41] Short NJ, Rytting ME, Cortes JE (2018). Acute myeloid leukaemia. Lancet.

[42] Döhner H (2017). Diagnosis and management of AML in adults: 2017 ELN recommendations from an international expert panel. Blood.

[43] Saultz J, Garzon R (2016). Acute myeloid leukemia: a concise review. J. Clin. Med..

[44] Di Nardo CD, Cortes JE (2016). Mutations in AML: prognostic and therapeutic implications. Hematology.

[45] Stone RM (2017). Midostaurin plus chemotherapy for acute myeloid leukemia with a FLT3 mutation. N. Engl. J. Med..

[46] Andreeff M (1999). Expression of Bcl-2-related genes in normal and AML progenitors: changes induced by chemotherapy and retinoic acid. Leukemia.

[47] Letai A, Sorcinelli MD, Beard C, Korsmeyer SJ (2004). Antiapoptotic BCL-2 is required for maintenance of a model leukemia. Cancer Cell..

[48] Zhou JD (2019). BCL2 overexpression: clinical implication and biological insights in acute myeloid leukemia. Diagn. Pathol..

[49] Bensi L (1995). Bcl-2 oncoprotein expression in acute myeloid leukemia. Haematologica.

[50] Lauria F (1997). High BCL-2 expression in acute myeloid leukemia cells correlates with CD34 positivity and complete remission rate. Leukemia.

[51] Wang J (2000). Cell permeable Bcl-2 binding peptides: a chemical approach to apoptosis induction in tumor cells. Cancer Res..

[52] Wang JL (2000). Structure-based discovery of an organic compound that binds Bcl-2 protein and induces apoptosis of tumor cells. Proc. Natl Acad. Sci. USA.

[53] Pallis M, Zhu YM, Russell NH (1997). Bcl-x(L) is heterogeneously expressed by acute myeloblastic leukaemia cells and is associated with autonomous growth in vitro and with P-glycoprotein expression. Leukemia.

[54] Konopleva M (2002). The anti-apoptotic genes Bcl-X(L) and Bcl-2 are over-expressed and contribute to chemoresistance of non-proliferating leukaemic CD34+ cells. Br. J. Haematol..

[55] Kaufmann SH (1998). Elevated expression of the apoptotic regulator Mcl-1 at the time of leukemic relapse. Blood.

[56] Xiang Z (2010). Mcl1 haploinsufficiency protects mice from Myc-induced acute myeloid leukemia. J. Clin. Invest..

[57] Glaser SP (2012). Anti-apoptotic Mcl-1 is essential for the development and sustained growth of acute myeloid leukemia. Genes Dev..

[58] Yoshimoto G (2009). FLT3-ITD up-regulates MCL-1 to promote survival of stem cells in acute myeloid leukemia via FLT3-ITD-specific STAT5 activation. Blood.

[59] Wei AH (2020). Targeting MCL-1 in hematologic malignancies: rationale and progress. Blood Rev..

[60] Ryan J, Montero J, Rocco J, Letai A (2016). iBH3: simple, fixable BH3 profiling to determine apoptotic priming in primary tissue by flow cytometry. Biol. Chem..

[61] Ryan J, Letai A (2013). BH3 profiling in whole cells by fluorimeter or FACS. Methods.

[62] Lessene G, Czabotar PE, Colman PM (2008). BCL-2 family antagonists for cancer therapy. Nat. Rev. Drug Discov..

[63] Leverson JD (2017). Found in translation: how preclinical research is guiding the clinical development of the BCL2-selective inhibitor venetoclax. Cancer Discov..

[64] Oltersdorf T (2005). An inhibitor of Bcl-2 family proteins induces regression of solid tumours. Nature.

[65] Tse C (2008). ABT-263: a potent and orally bioavailable Bcl-2 family inhibitor. Cancer Res..

[66] Zhang H (2007). Bcl-2 family proteins are essential for platelet survival. Cell Death Differ..

[67] Roberts AW (2012). Substantial susceptibility of chronic lymphocytic leukemia to BCL2 inhibition: results of a phase I study of navitoclax in patients with relapsed or refractory disease. J. Clin. Oncol..

[68] Souers AJ (2013). ABT-199, a potent and selective BCL-2 inhibitor, achieves antitumor activity while sparing platelets. Nat. Med..

[69] Choudhary GS (2015). MCL-1 and BCL-xL-dependent resistance to the BCL-2 inhibitor ABT-199 can be overcome by preventing PI3K/AKT/mTOR activation in lymphoid malignancies. Cell Death Dis..

[70] Lin KH (2016). Targeting MCL-1/BCL-XL forestalls the acquisition of resistance to ABT-199 in acute myeloid leukemia. Sci. Rep..

[71] Niu X (2016). Binding of released Bim to Mcl-1 is a mechanism of intrinsic resistance to ABT-199 which can be overcome by combination with daunorubicin or cytarabine in AML cells. Clin. Cancer Res..

[72] Vo TT (2012). Relative mitochondrial priming of myeloblasts and normal HSCs determines chemotherapeutic success in AML. Cell.

[73] Schoenwaelder SM (2011). Bcl-xL-inhibitory BH3 mimetics can induce a transient thrombocytopathy that undermines the hemostatic function of platelets. Blood.

[74] Wang X (2013). Deletion of MCL-1 causes lethal cardiac failure and mitochondrial dysfunction. Genes Dev..

[75] Thomas RL (2013). Loss of MCL-1 leads to impaired autophagy and rapid development of heart failure. Genes Dev..

[76] Graham SM (2002). Primitive, quiescent, Philadelphia-positive stem cells from patients with chronic myeloid leukemia are insensitive to STI571 in vitro. Blood.

[77] Holyoake TL, Vetrie D (2017). The chronic myeloid leukemia stem cell: stemming the tide of persistence. Blood.

[78] Ng KP (2012). A common BIM deletion polymorphism mediates intrinsic resistance and inferior responses to tyrosine kinase inhibitors in cancer. Nat. Med..

[79] La Rosée P, Deininger MW (2010). Resistance to imatinib: mutations and beyond. Semin Hematol..

[80] Ko TK, CTHH Chuah, JWJJ Huang (2014). Ng K-PP, Ong ST. The BCL2 inhibitor ABT-199 significantly enhances imatinib-induced cell death in chronic myeloid leukemia progenitors. Oncotarget.

[81] Mak DH (2012). Activation of apoptosis signaling eliminates CD34+ progenitor cells in blast crisis CML independent of response to tyrosine kinase inhibitors. Leukemia.

[82] Airiau K (2012). ABT-737 increases tyrosine kinase inhibitor-induced apoptosis in chronic myeloid leukemia cells through XIAP downregulation and sensitizes CD34+ CD38- population to imatinib. Exp. Hematol..

[83] Dasatinib and venetoclax in treating patients with Philadelphia chromosome positive or BCR-ABL1 positive early chronic phase chronic myelogenous leukemia. https://clinicaltrials.gov/ct2/show/NCT02689440 (2020).

[84] Decitabine, venetoclax, and ponatinib for the treatment of Philadelphia chromosome-positive acute myeloid leukemia or myeloid blast phase chronic myelogenous leukemia https://clinicaltrials.gov/ct2/show/NCT04188405 (2020).

[85] Niu X (2014). Acute myeloid leukemia cells harboring MLL fusion genes or with the acute promyelocytic leukemia phenotype are sensitive to the Bcl-2-selective inhibitor ABT-199. Leukemia.

[86] Chan SM (2015). Isocitrate dehydrogenase 1 and 2 mutations induce BCL-2 dependence in acute myeloid leukemia. Nat. Med..

[87] A phase 2 study of ABT-199 in subjects with acute myelogenous leukemia (AML) https://clinicaltrials.gov/ct2/show/NCT01994837 (2021).

[88] Konopleva M (2016). Efficacy and biological correlates of response in a phase II study of venetoclax monotherapy in patients with acute myelogenous leukemia. Cancer Discov..

[89] Pollyea DA, Amaya M, Strati P, Konopleva MY (2019). Venetoclax for AML: changing the treatment paradigm. Blood Adv..

[90] Stoetzer OJ (1996). Association of BCL-2, BAX, BCL-xL and interleukin-1 beta-converting enzyme expression with initial response to chemotherapy in acute myeloid leukemia. Leukemia.

[91] Karakas T (1998). High expression of BCL-2 mRNA as a determinant of poor prognosis in acute myeloid leukemia. Ann. Oncol..

[92] Bogenberger JM (2014). BCL-2 family proteins as 5-azacytidine-sensitizing targets and determinants of response in myeloid malignancies. Leukemia.

[93] Bogenberger JM (2015). Ex vivo activity of BCL-2 family inhibitors ABT-199 and ABT-737 combined with 5-azacytidine in myeloid malignancies. Leuk. Lymphoma.

[94] Tsao T (2012). Concomitant inhibition of DNA methyltransferase and BCL-2 protein function synergistically induce mitochondrial apoptosis in acute myelogenous leukemia cells. Ann. Hematol..

[95] Study of ABT-199 (GDC-0199) in combination with azacitidine or decitabine (chemo combo) in subjects with acute myelogenous leukemia (AML). https://clinicaltrials.gov/ct2/show/NCT02203773 (2021).

[96] DiNardo CD (2018). Safety and preliminary efficacy of venetoclax with decitabine or azacitidine in elderly patients with previously untreated acute myeloid leukaemia: a non-randomised, open-label, phase 1b study. Lancet Oncol..

[97] DiNardo CD (2019). Venetoclax combined with decitabine or azacitidine in treatment-naive, elderly patients with acute myeloid leukemia. Blood.

[98] A study of venetoclax in combination with azacitidine versus azacitidine in treatment naïve subjects with acute myeloid leukemia who are ineligible for standard induction therapy https://clinicaltrials.gov/ct2/show/NCT02993523 (2021).

[99] DiNardo CD (2020). Azacitidine and venetoclax in previously untreated acute myeloid leukemia. N. Engl. J. Med..

[100] Benedict WF, Harris N, Karon M (1970). Kinetics of 1-β-d-arabinofuranosylcytosine-induced chromosome breaks. Cancer Res..

[101] Keith FJ, Bradbury DA, Zhu YM, Russell NH (1995). Inhibition of BCL-2 with antisense oligonucleotides induces apoptosis and increases the sensitivity of AML blasts to Ara-C. Leukemia.

[102] Xie C (2015). Obatoclax potentiates the cytotoxic effect of cytarabine on acute myeloid leukemia cells by enhancing DNA damage. Mol. Oncol..

[103] A study evaluating venetoclax in combination with low-dose cytarabine in treatment-naïve participants with acute myelogenous leukemia https://clinicaltrials.gov/ct2/show/NCT02287233 (2021).

[104] Wei AH (2019). Venetoclax combined with low-dose cytarabine for previously untreated patients with acute myeloid leukemia: results from a phase Ib/II study. J. Clin. Oncol..

[105] A study of venetoclax in combination with low dose cytarabine versus low dose cytarabine alone in treatment naive patients with acute myeloid leukemia who are ineligible for intensive chemotherapy. https://clinicaltrials.gov/ct2/show/NCT03069352 (2021).

[106] Wei AH (2020). Venetoclax plus LDAC for newly diagnosed AML ineligible for intensive chemotherapy: a phase 3 randomized placebo-controlled trial. Blood.

[107] Ma J (2019). Inhibition of Bcl-2 synergistically enhances the antileukemic activity of midostaurin and gilteritinib in preclinical models of FLT3-mutated acute myeloid leukemia. Clin. Cancer Res..

[108] Seipel K, Marques MAT, Sidler C, Mueller BU, Pabst T (2018). MDM2- and FLT3-inhibitors in the treatment of FLT3-ITD acute myeloid leukemia, specificity and efficacy of NVP-HDM201 and midostaurin. Haematologica.

[109] Zhang W (2008). Sorafenib induces apoptosis of AML cells via Bim-mediated activation of the intrinsic apoptotic pathway. Leukemia.

[110] Rahmani M, Davis EM, Bauer C, Dent P, Grant S (2005). Apoptosis induced by the kinase inhibitor BAY 43-9006 in human leukemia cells involves down-regulation of Mcl-1 through inhibition of translation. J. Biol. Chem..

[111] Rahmani M (2012). Inhibition of Bcl-2 antiapoptotic members by obatoclax potently enhances sorafenib-induced apoptosis in human myeloid leukemia cells through a Bim-dependent process. Blood.

[112] Yang J, Ikezoe T, Nishioka C, Yokoyama A (2013). Over-expression of Mcl-1 impairs the ability of ATRA to induce growth arrest and differentiation in acute promyelocytic leukemia cells. Apoptosis.

[113] Perri M (2014). BCL-xL/MCL-1 inhibition and RARγ antagonism work cooperatively in human HL60 leukemia cells. Exp. Cell Res..

[114] Jaffrézou JP (1996). Daunorubicin-induced apoptosis: triggering of ceramide generation through sphingomyelin hydrolysis. EMBO J..

[115] Kim YH, Park JW, Lee JY, Surh YJ, Kwon TK (2003). Bcl-2 overexpression prevents daunorubicin-induced apoptosis through inhibition of XIAP and Akt degradation. Biochem. Pharmacol..

[116] Allouche M (1997). Influence of Bcl-2 overexpression on the ceramide pathway in daunorubicin-induced apoptosis of leukemic cells. Oncogene.

[117] Dariushnejad H (2014). ABT-737, synergistically enhances daunorubicin-mediated apoptosis in acute myeloid leukemia cell lines. Adv. Pharm. Bull..

[118] Moujalled DM (2019). Combining BH3-mimetics to target both BCL-2 and MCL1 has potent activity in pre-clinical models of acute myeloid leukemia. Leukemia.

[119] Carter BZ (2019). TP53 deficient/mutant AMLs are resistant to individual BH3 mimetics: high efficacy of combined inhibition of Bcl-2 and Mcl-1. Blood.

[120] Yecies D, Carlson NE, Deng J, Letai A (2010). Acquired resistance to ABT-737 in lymphoma cells that up-regulate MCL-1 and BFL-1. Blood.

[121] Fresquet V, Rieger M, Carolis C, García-Barchino MJ, Martinez-Climent JA (2014). Acquired mutations in BCL2 family proteins conferring resistance to the BH3 mimetic ABT-199 in lymphoma. Blood.

[122] Hormi M (2020). Pairing MCL‐1 inhibition with venetoclax improves therapeutic efficiency of BH3‐mimetics in AML. Eur. J. Haematol..

[123] Wan Y, Dai N, Tang Z, Fang H (2018). Small-molecule Mcl-1 inhibitors: emerging anti-tumor agents. Eur. J. Med. Chem..

[124] Szlávik Z (2019). Structure-guided discovery of a selective MCL-1 inhibitor with cellular activity. J. Med. Chem..

[125] Yi X (2020). AMG-176, an Mcl-1 antagonist, shows preclinical efficacy in chronic lymphocytic leukemia. Clin. Cancer Res..

[126] Hird AW, Tron AE (2019). Recent advances in the development of Mcl-1 inhibitors for cancer therapy. Pharm. Ther..

[127] Tron AE (2018). Discovery of Mcl-1-specific inhibitor AZD5991 and preclinical activity in multiple myeloma and acute myeloid leukemia. Nat. Commun..

[128] Phase I study of S64315 administred intravenously in patients with acute myeloid leukaemia or myelodysplastic syndrome. https://clinicaltrials.gov/ct2/show/NCT02979366 (2020).

[129] AMG 176 first in human trial in subjects with relapsed or refractory multiple myeloma and subjects with relapsed or refractory acute myeloid leukemia. https://clinicaltrials.gov/ct2/show/NCT02675452 (2020).

[130] Safety, tolerability, pharmacokinetics and efficacy of AMG 397 in subjects with multiple myeloma, NHL, and AML. https://clinicaltrials.gov/ct2/show/NCT03465540 (2020).

[131] Study of AZD5991 in relapsed or refractory haematologic malignancies. https://clinicaltrials.gov/ct2/show/NCT03218683 (2021).

[132] Phase I dose escalation study of intravenously administered S64315 in combination with orally administered venetoclax in patients with acute myeloid leukaemia. https://clinicaltrials.gov/ct2/show/NCT03672695 (2020).

[133] A study of venetoclax and AMG 176 in patients with relapsed/refractory hematologic malignancies. https://clinicaltrials.gov/ct2/show/NCT03797261 (2020).

[134] Dasmahapatra G, Almenara JA, Grant S (2006). Flavopiridol and histone deacetylase inhibitors promote mitochondrial injury and cell death in human leukemia cells that overexpress Bcl-2. Mol. Pharmacol..

[135] Baker A (2016). The CDK9 inhibitor dinaciclib exerts potent apoptotic and antitumor effects in preclinical models of MLL-rearranged acute myeloid leukemia. Cancer Res..

[136] Chantkran W, Zheleva D, Frame S, Hsieh Y-C, Copland M (2019). Combination of CYC065, a second generation CDK2/9 inhibitor, with venetoclax or standard chemotherapies - a novel therapeutic approach for acute myeloid leukaemia (AML). Blood.

[137] Cidado J (2020). AZD4573 is a highly selective CDK9 inhibitor that suppresses Mcl-1 and induces apoptosis in hematologic cancer cells. Clin. Cancer Res..

[138] Ivosidenib and venetoclax with or without azacitidine in treating participants with IDH1 mutated hematologic malignancies. https://clinicaltrials.gov/ct2/show/NCT03471260?term=bh3+profiling&draw=2&rank=8 (2020).

[139] Venetoclax with combination chemotherapy in treating patients with newly diagnosed or relapsed or refractory acute myeloid leukemia. https://clinicaltrials.gov/ct2/show/NCT03214562?term=bh3+profiling&draw=2&rank=6 (2020).

[140] A phase 1b/2 study of alvocidib plus decitabine or azacitidine in patients with MDS. https://clinicaltrials.gov/ct2/show/NCT03593915?term=bh3+profiling&draw=2&rank=2 (2020).

[141] Venetoclax and decitabine in treating participants with relapsed/refractory acute myeloid leukemia or relapsed high-risk myelodysplastic syndrome. https://clinicaltrials.gov/ct2/show/NCT03404193?term=bh3+profiling&draw=2&rank=5 (2020).

[142] Ibrutinib and venetoclax in treating patients with chronic lymphocytic leukemia after ibrutinib resistance. https://clinicaltrials.gov/ct2/show/NCT03943342?term=bh3+profiling&draw=2&rank=7 (2020).

[143] Everolimus with multiagent re-induction chemotherapy in pediatric patients with ALL. https://clinicaltrials.gov/ct2/show/NCT01523977?term=bh3+profiling&draw=2&rank=4 (2020).

[144] Ishizawa J (2015). Mitochondrial profiling of acute myeloid leukemia in the assessment of response to apoptosis modulating drugs. PLoS ONE.

[145] Alvocidib Biomarker-driven Phase 2 AML Study. https://clinicaltrials.gov/ct2/show/NCT02520011?term=NCT02520011&draw=2&rank=1 (2020).

[146] Punnoose EA (2016). Expression profile of BCL-2, BCL-XL, and MCL-1 predicts pharmacological response to the BCL-2 selective antagonist venetoclax in multiple myeloma models. Mol. Cancer Ther..

[147] Soderquist RS (2018). Systematic mapping of BCL-2 gene dependencies in cancer reveals molecular determinants of BH3 mimetic sensitivity. Nat. Commun..

[148] Iavarone C (2019). Combined MEK and Bcl-2/XL inhibition is effective in high-grade serous ovarian cancer patient–derived xenograft models and BIM levels are predictive of responsiveness. Mol. Cancer Ther..

